# The Relationship between Visual Field Index and Estimated Number of Retinal Ganglion Cells in Glaucoma

**DOI:** 10.1371/journal.pone.0076590

**Published:** 2013-10-16

**Authors:** Amir H. Marvasti, Andrew J. Tatham, Linda M. Zangwill, Christopher A. Girkin, Jeffrey M. Liebmann, Robert N. Weinreb, Felipe A. Medeiros

**Affiliations:** 1 Hamilton Glaucoma Center, Department of Ophthalmology, University of California San Diego, San Diego, California, United States of America; 2 Boston University School of Medicine, Boston, Massachusetts, United States of America; 3 Department of Ophthalmology, University of Alabama, Birmingham, Alabama, United States of America; 4 New York Eye and Ear Infirmary, New York, New York, United States of America; University of Rochester, United States of America

## Abstract

**Purpose:**

To evaluate the relationship between visual field index (VFI) and the estimated number of retinal ganglion cells (RGCs) in glaucoma.

**Methods:**

A multicenter study of 1,245 healthy, glaucomatous and suspected glaucomatous eyes of 1,245 subjects recruited from the Diagnostic Innovations in Glaucoma Study (DIGS) and African Descent and Glaucoma Evaluation Study (ADAGES). All eyes underwent standard automated perimetry (SAP) and time-domain optical coherence tomography (TD-OCT). Estimates of RGC count and percentage of RGCs remaining, compared to age-matched healthy eyes, were calculated from TD-OCT using a previously described formula. Smoothing spline curves were fitted to examine the relationship between VFI and the percent remaining RGCs. The first derivative (i.e., slopes) of these curves was used to explore the relationship between changes in these measures.

**Results:**

The relationships between the VFI and both estimated RGC counts and the percent remaining RGCs were nonlinear. A unit number of VFI loss corresponded to substantially greater loss of estimated RGCs and estimated percentage of RGCs remaining in early compared to late disease.

**Conclusions:**

The relationship between VFI and estimated RGC counts is nonlinear and the index substantially underestimates the amount of neural loss early in the disease. Disease severity should be taken into account when interpreting rates of VFI change over time.

## Introduction

Glaucoma is a progressive optic neuropathy, characterized by loss of retinal ganglion cells (RGCs), and associated morphological changes to the optic nerve and retinal nerve fiber layer (RNFL). [1] Structural damage is usually accompanied by decrease in visual function, which ultimately can lead to blindness. A major challenge in the management of glaucoma is how to best determine severity of disease and estimate the rate of progression. [2], [3] Standard automated perimetry (SAP) is commonly used for these purposes, for example, using summary global SAP indices, such as mean deviation (MD) and visual field index (VFI) [4]–[7].

The VFI, introduced by Bengtsson and Heijl, expresses visual function as a percentage of normal age-corrected sensitivity. [7] Therefore, the VFI of an eye with a completely normal visual field is 100% and the VFI of a perimetrically blind eye is 0%. The VFI is automatically computed in the current “Statpac software” of the Humphrey Field Analyzer (HFA II; Carl Zeiss Meditec, Inc., Dublin, California, USA). Change in VFI is also used to estimate the rate of progression using so-called “trend-based” analysis. [4]–[6] A trend-based analysis using a linear regression analysis of VFI values over time is calculated as part of the Guided Progression Analysis (GPA), from which the patient’s rate of progression, in percent VFI loss per year, can be determined [8].

The VFI was originally developed to address the shortcomings of MD, and compared to MD, VFI is thought to be less affected by the confounding effects of media opacities, such as cataract. [7] The VFI also includes a weighting, whereby central visual field test points are assigned greater significance than those located more peripherally. The weighting is based on cortical magnification and reflects the higher density of RGCs in the macula. It is claimed that by using this weighting procedure, the VFI would more closely reflect underlying loss of RGCs [7].

Although direct counting of RGCs is not yet possible *in vivo*, empirical formulas have recently been described which allow estimation of the number of RGCs from optical coherence tomography (OCT) RNFL thickness measurements. [9] Estimates derived using these formulas have shown good correlation to histologic RGC counts [9] and have demonstrated good ability for staging disease. [10]–[12] The aim of the present study was to evaluate the relationship between VFI and estimated numbers of RGCs obtained from OCT data in the same subjects. The study of this relationship may provide us with a better understanding of the properties of the VFI and its suitability as a surrogate for neural losses in glaucoma.

## Methods

### Ethics Statement

Written informed consent was obtained from all participants, and the institutional review boards and human subjects committees of all 3 sites (University of California, San Diego; New York Eye and Ear Infirmary; and University of Alabama at Birmingham) approved all of the methods. All methods adhered to the tenets of the Declaration of Helsinki for research involving human subjects.

### Description of Study Population

This was a cross-sectional study involving participants from 2 prospective longitudinal studies designed to evaluate optic nerve structure and visual function in glaucoma; the African Descent and Glaucoma Evaluation Study (ADAGES) and the Diagnostic Innovations in Glaucoma Study (DIGS). The 3-site ADAGES collaboration includes the Hamilton Glaucoma Center at the Department of Ophthalmology, University of California, San Diego (UCSD) (data coordinating center); the New York Eye and Ear Infirmary; and the Department of Ophthalmology, University of Alabama at Birmingham (UAB). Although the DIGS includes only patients recruited at UCSD, the protocols of the two studies are identical. Methodological details have been described previously [13].

At each visit during follow-up subjects underwent a comprehensive ophthalmologic examination including review of medical history, best-corrected visual acuity, slit-lamp biomicroscopy, intraocular pressure (IOP) measurement, gonioscopy, dilated fundoscopic examination, stereoscopic optic disc photography, and automated perimetry using the Swedish interactive threshold algorithm (SITA Standard 24-2). Only subjects with open angles on gonioscopy were included. Subjects were excluded if they presented with a best-corrected visual acuity less than 20/40, spherical refraction outside ±5.0 diopters and/or cylinder correction outside 3.0 diopters, or any other ocular or systemic disease that could affect the optic nerve or the visual field.

The study included 1245 eyes of 1245 subjects. Of the 1245 eyes, 438 (35%) had glaucomatous visual field defects, 239 (19%) had glaucomatous optic neuropathy (GON) without visual field abnormalities, 234 (19%) had ocular hypertension (OHT) and 334 (28%) were healthy. A glaucomatous visual field was defined by the presence of a repeatable (≥2 consecutive) abnormal visual field test result on the 24-2 program of the Humphrey visual field analyzer (Carl Zeiss Meditec, Inc., Dublin, California, USA). An abnormal visual field result was defined as having a pattern standard deviation outside the 95% confidence limits or a glaucoma hemifield test (GHT) result outside the reference range. Glaucomatous optic neuropathy was defined by the presence of neuroretinal rim thinning or RNFL defects on masked stereophotograph assessment. OHT was defined as intraocular pressure (IOP) greater than 21 mmHg in the presence of a healthy-appearing optic disc without a repeatable abnormal visual field result. Healthy subjects were recruited from the general population through advertisements. Healthy eyes had intraocular pressure of 21 mmHg or less with no history of increased IOP and no visual field abnormalities.

### Standard Automated Perimetry

All patients underwent SAP testing using the SITA-standard 24-2 strategy within 30 days of imaging. All visual fields were evaluated by the UCSD Visual Field Assessment Center (VisFACT). [14] Visual fields with more than 33% fixation losses or false-negative errors, or more than 15% false-positive errors were excluded. The only exception was the inclusion of visual fields with false-negative errors of more than 33% when the field showed advanced disease (MD worse than −12 dB). Visual fields exhibiting a learning effect (i.e., initial tests showing consistent improvement on visual field indexes) were also excluded. Visual fields were further reviewed for the following artifacts: lid and rim artifacts; fatigue effects; inappropriate fixation; evidence that the visual field results were due to a disease other than glaucoma (such as homonymous hemianopia); and inattention. The VisFACT requested repeats of unreliable visual field test results and these were obtained whenever possible.

### Optical Coherence Tomography

Subjects underwent time domain-OCT (TD-OCT) with dilated pupils using the Stratus OCT (Carl Zeiss Meditec, Inc., Dublin, California, USA). [15] The fast RNFL algorithm was used to obtain RNFL thickness measurements. Three images were acquired from each subject, with each image consisting of 256 A-scans along a 3.4-mm diameter circular ring around the optic disc. The average parapapillary RNFL thickness (360-degree measure) was calculated automatically by the software and was used in the study. RNFL scans were also evaluated as to the adequacy of the segmentation algorithm for detection of the RNFL. Only scans without overt RNFL segmentation failure were included in the study. Quality assessment of Stratus OCT scans was undertaken by an experienced examiner masked to the results of the other tests. Good-quality scans had to have a focused image of the ocular fundus, signal strength of more than 7, and the presence of a centered circular ring around the optic disc.

### Retinal Ganglion Cell Estimation

Estimates of RGC counts were obtained using formulas based on previous work by Harwerth and colleagues [9] on the development and validation of a model-linking structure and function in glaucoma. Based on experimental studies in monkeys, the authors derived an empirical model relating measurements in OCT to histological RGC counts as a function of RNFL thickness. The model considered the effect of aging in the axonal density and the effect of disease severity on the relationship between the neuronal and non-neuronal components of the RNFL thickness estimates obtained by OCT. To derive the total number of RGC axons from the global RNFL thickness measurement obtained by OCT (RGC), one can apply the following formulas:
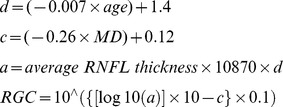



In the above formulas, *d* corresponds to the axonal density (axons/ µm^2^), *c* is a correction factor for the severity of disease to take into account remodeling of the RNFL axonal and non-axonal composition. The variable *a* corresponds to the number of axons passing toward the optic disc at the point of the OCT circumference.

After estimates of RGC were obtained, a linear regression model was developed to relate the estimated number of RGCs to age and optic disc area in the healthy population. The purpose was to develop a model to predict the expected number of RGCs according to age and optic disc area. To avoid model overfitting, the regression parameters were obtained using only half of the normal eyes (development sample). After the expected number of RGCs was calculated for each eye, the estimated percentage of remaining RGCs was obtained by dividing the estimated number of RGCs by age-corrected expected RGC estimates:
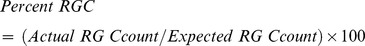



### Statistical Analysis

The primary purpose of the analysis was to quantify the relationship between VFI and estimated RGC counts as assessed from cross-sectional data. Scatter plots were constructed of VFI versus the estimated number of RGCs and cubic smooth spline curves used to explore the relationship between the variables. A spline function is a curve constructed from polynomial segments that are subject to continuity at their joints. The first derivatives (i.e., slopes) of these curves were calculated to examine the relationship between changes in VFI and change in estimated RGC counts.

As VFI is an age-corrected index, its relationship with estimated RGC counts, which does not take age into account, might be confounded. For example, both normal elderly and normal younger subjects might have a VFI value of 100% but the elderly subject will have lower RGC counts as part of the physiological loss of RGCs that occurs with aging. [16] To account for this, using expected RGC values in a normal population, we calculated the age-corrected percent of RGCs remaining (PercentRGC) in each subject. Cubic smoothing spline curves and first derivatives were also calculated to investigate the relationship between VFI and PercentRGC.

All statistical analyses were performed with commercially available software (Stata 11; StataCorp, College Station, TX). The alpha level (type I error) was set at 0.05.

## Results

**Figure 1:**
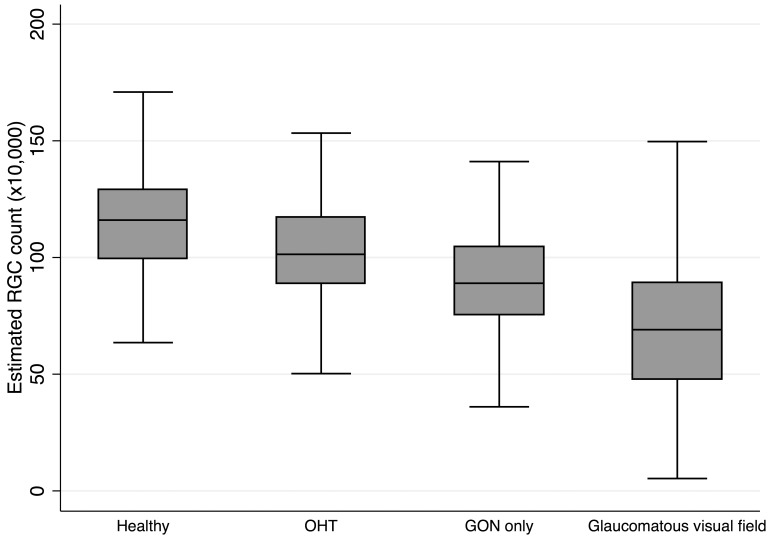
Boxplot illustrating the distribution of estimates of retinal ganglion cell (RGC) counts in the four diagnostic categories. The diagnostic categories include: healthy eyes, eyes with ocular hypertension (OHT), eyes with GON but normal visual fields (GON only), and eyes with glaucomatous visual fields.

The mean (± standard deviation) age of subjects included in the study was 58±15 years. Table 1 shows demographic and clinical characteristics of the eyes included in the study. The mean (± standard deviation) VFI was 99.1±1.2% in healthy eyes, 99.1±1.4% in eyes with OHT, 98.7±2.4% in eyes with GON but normal visual fields and 87.5±16.7% in eyes with perimetric glaucoma, compared to an estimated percent of remaining RGCs (PercentRGC) of 95.1±7.3%, 93.4±8.7%, 85.1±13.5%, and 66.9±24.6%, respectively. Figure 1 shows boxplots of the distribution of estimated RGC counts in each of the groups.

**Table 1:** Clinical and Demographic Characteristics (mean ± standard deviation) according to the Diagnostic Category.

	Healthy(n = 334 eyes)	OHT(n = 234 eyes)	GON with Normal VF(n = 239 eyes)	Glaucomatous VF loss(n = 438 eyes)
**Age (yrs)**	48±15	59±13	62±13	63±13
**Sex, female**	209 (63%)	140 (60%)	150 (63%)	240 (55%)
**Race**				
**Caucasian**	157 (47%)	141 (60%)	145 (61%)	205 (47%)
**African-American**	172 (52%)	87 (37%)	85 (36%)	219 (50%)
**Other**	5 (1%)	6 (3%)	9 (4%)	14 (3%)
**MD (dB)**	−0.2±1.4	−0.1±1.4	−0.6±1.7	−5.0±5.7
**PSD (dB)**	1.6±0.4	1.6±0.6	1.8±0.9	5.1±3.7
**VFI (%)**	99.1±1.2	99.1±1.4	98.7±2.4	87.5±16.7
**Estimated number of RGCs (cells)**	1,149,788±209,240	1,013,631±189,069	895,235±206,495	687,219±293,653
**PercentRGC (%)**	95.1±7.3	93.4±8.7	85.1±13.5	66.9±24.6

MD = mean deviation.

PSD = pattern standard deviation.

VFI = visual field index.

RGC = retinal ganglion cell.

PercentRGC = estimated percent of RGCs remaining.

**Figure 2:**
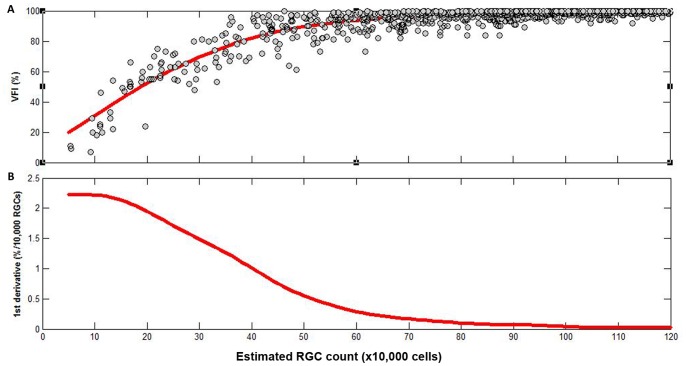
Relationship between visual field index (VFI) and retinal ganglion cells (RGC). A. Relationship between visual field index (VFI) and estimated retinal ganglion cell (RGC) counts. B. First derivatives of the curve shown in Figure 2A plotted against estimated RGC counts. The derivatives indicate the amount of change in VFI per 10,000 RGCs at different levels of estimated number of RGCs.

Figure 2A shows the relationship between VFI and estimated RGC counts. A curve was fit to the data using smoothing splines. Figure 2B shows the first derivatives obtained from the curve shown in Figure 2A, plotted against the estimated number of RGCs. The derivatives correspond to the slopes of the curve shown in Figure 2A at different values of estimated RGC count. The derivatives indicate the amount of change in VFI expected for a 10,000 cell change in the estimated number of RGCs. For example, for an eye with an estimated RGC count of 400,000 cells, an additional loss of 10,000 RGCs would correspond to a change in VFI of approximately 1%. Figure 2B demonstrates that the amount of change in VFI for a change in estimated number of RGCs varies according to RGC count (i.e., with disease severity). Alternatively, loss of a given number of RGCs would be expected to correspond to largely different amounts of change in VFI depending on the stage of disease.

Table 2 summarizes the expected changes in VFI that would occur for different amounts of RGC losses according to the stage of disease. For example, for an eye with an estimated RGC count of 1,150,000 cells and VFI of 100%, which corresponded to the median value in healthy eyes, a loss of 10,000 RGCs would be expected to correspond to a very small change in VFI, of only 0.03%. In an eye with 1,150,000 RGCs and VFI of 100%, even a very large loss of 100,000 RGCs would still correspond to only a 0.3% change in VFI. In contrast, in an eye with severe glaucomatous damage, with an estimated RGC count of 240,000 cells and VFI of 60%, a loss of 10,000 RGCs would correspond to a change in VFI of 1.9%. A loss of 100,000 RGCs would correspond to a very large change in VFI of 19.8%.

**Table 2:** Change in standard automated perimetry (SAP) visual field index (VFI) corresponding to different amounts of change in estimated retinal ganglion cell (RGC) counts at different stages of disease.

Stage of disease		Change in VFI (%) for a change of:	
VFI (%)	Estimated RGC count (cells)	10,000 RGCs	100,000 RGCs
**100**	1,150,000	0.03	0.3
**95**	650,000	0.2	3.0
**90**	510,000	0.6	7.2
**80**	380,000	1.1	13.5
**60**	240,000	1.9	19.8
**40**	140,000	2.3	22.1

VFI = visual field index.

RGC = retinal ganglion cell.

**Figure 3:**
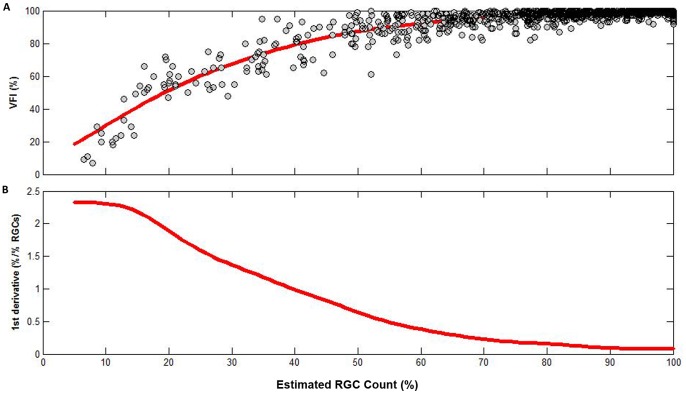
Relationship between visual field index (VFI) and remaining retinal ganglion cells (RGC). A. Relationship between visual field index (VFI) and age-corrected percent of remaining estimated retinal ganglion cell (RGC) counts. B. First derivatives of the curve shown on Figure 3A plotted against the age-corrected percent of remaining estimated RGC counts (B). The derivative indicates the amount of change in VFI per 1% of the remaining RGC counts at different levels of remaining estimated number of RGCs (%).

**Figure 4:**
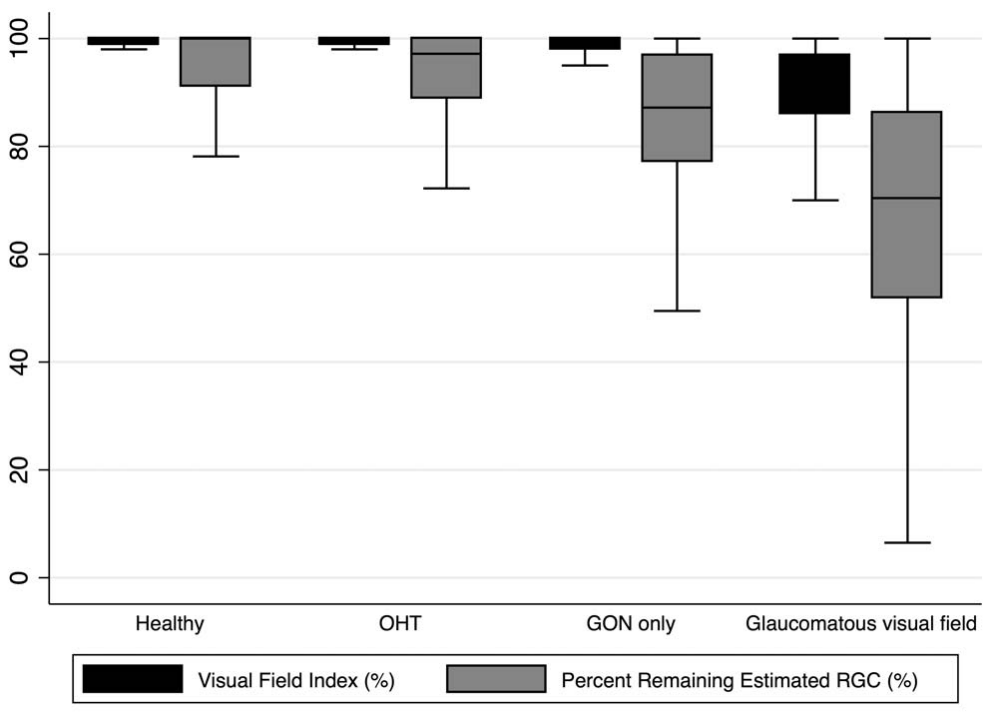
Boxplot comparing the distribution of visual field index (VFI) and age-corrected percent of remaining estimated retinal ganglion cell (RGC) counts in the four diagnostic categories. The diagnostic categories include: healthy eyes, eyes with ocular hypertension (OHT), eyes with GON but normal visual fields (GON only), and eyes with glaucomatous visual fields.

The relationship between VFI and the age-corrected percent of RGCs remaining (PercentRGC) is shown in Figure 3A. A curve was fit to the data using smoothing splines. Figure 3B shows the first derivatives obtained from the curve shown on Figure 3A and plotted against the estimate of percent of RGCs remaining. Here the first derivatives indicate the amount of change in VFI for a 1% change in RGCs. For example, for an eye with an estimated 70% of RGCs remaining, an additional loss of 1% of RGCs would correspond to a change in VFI of approximately 0.25%. Figure 3B shows that the change in VFI for a given change in PercentRGC varies according to the estimated percent of RGCs remaining (i.e., with disease severity). This relationship is very similar to the relationship between VFI and estimated RGC counts, as demonstrated above. Figure 4 shows the distribution of VFI and PercentRGC in each one of the diagnostic groups.

## Discussion

In the present study, empirical formulas were used to estimate RGC counts from structural measurements in order to better understand the role of the VFI in the assessment of neural losses from glaucoma. The results demonstrated that the VFI does not have a linear relationship with estimated RGC counts. Changes in VFI corresponded to largely different amounts of estimated neural losses according to the severity of the disease. This result has significant implications for the use of VFI as a surrogate for neural losses in glaucoma and as an index for evaluation of progression and rates of change in the disease.

Given that the methodology for calculating VFI takes into account the higher density of RGCs in the central fovea, it has been proposed that the VFI would more closely reflect RGC losses than other visual field indices. [7] However, figures 2 and 3 demonstrate that the relationship between VFI and the absolute number of RGCs, and between VFI and the percentage of remaining RGCs is nonlinear. This finding is similar to the nonlinear relationship between MD and estimated RGCs. [12] A given decrease in VFI corresponds to substantially greater loss of estimated RGCs in early compared to late disease. Such nonlinear relationship between VFI and RGCs indicates that face value interpretations of rates of VFI loss can be misleading. In other words, a 1% change in VFI does not represent the same amount of neural loss throughout the disease continuum. This has important implications on the interpretation of rates of VFI change as provided on visual field printouts. For example, one might consider a rate of change in VFI of 0.3%/year slow and insignificant. However, as Figure 2 and Table 2 demonstrate, a change of 0.3% in VFI in early stages of the disease (with initial VFI of 100%) would correspond to a loss of as much as 100,000 RGCs. The same amount of RGC loss would correspond to a VFI loss of 22.1% for an eye with severe damage (with initial VFI of 40%) (Table 2). Consequently, the same amount of neural loss could correspond to very small (0.3%) or very large (22.1%) degrees of VFI loss, depending on the stage of disease.

Another consequence of the nonlinearity between VFI and RGC counts is that rates of VFI loss over the full course of the disease are unlikely to be linear, unless glaucoma is associated with exponentially *decreasing* loss of RGCs over time. For example, consider an eye with initial RGC count of 1,000,000 that is showing a linear rate of loss of 100,000 RGCs per year. This eye would lose all RGCs in 10 years. However, analysis of rates of VFI change during the first year of the disease would indicate a rate of only approximately 0.3%/year (Table 2). Therefore, sole reliance on linear rates of VFI change in this situation could potentially lead to severe underestimation of the risk of functional impairment. Even if one recognizes the nonlinear relationship between VFI and RGCs and interprets VFI values accordingly, small rates of change in VFI in percent/year will be more difficult to detect due to the variability of measurements and, therefore, sole reliance on VFI for measurement of rates of change in early to moderate disease will still have the potential for underestimating neural losses. It is important to emphasize, however, that linear rates of visual field loss provided by VFI may still provide clinically relevant information with regard to the presence of change in visual function over time, especially in moderate to advanced damage and over relatively short periods of follow-up. Additionally, it should be recognized that, despite representing a smaller amount of neural loss, a given VFI percent loss in patients with severe damage would carry a higher risk of producing disability than the same VFI loss in those with normal visual fields or early visual field loss since the patient will be closer to functional impairment.

The observed discrepancy between VFI and percentage of remaining RGCs, and the nonlinear relationship between VFI and RGC loss could be explained by a number of reasons. Although given as a percentage, VFI is actually a summary index calculated based on visual field threshold sensitivities that are originally acquired and reported using a logarithmic decibel scale. An effect of the decibel scale is that losses are compressed in the top (early disease) of the scale while enlarged at the bottom (late disease). Therefore, significant structural changes in early disease may translate into relatively small perimetric changes. [17] VFI is also designed to be resistant to diffuse media opacity, which is beneficial in the presence of cataract, but at the cost of lower sensitivity to diffuse visual field changes that may be an essential component of visual field damage. There is evidence that diffuse visual field loss may occur in early glaucoma before the development of discrete nerve fiber-bundle defects. [18]–[20] In fact, a previous study has shown that the VFI can be close to its maximum value of 100% even in eyes with a MD as low as −5 dB. [21] Therefore, including only localized field loss in progression analysis may result in some level of underestimation of progression by missing global damage. This is especially relevant in early stages of the disease when focal losses may not yet be well established [21].

These findings collectively highlight the shortcomings of current perimetric techniques for estimation of neural losses in early glaucoma and have encouraged the development and use of imaging technologies for early detection and follow-up of glaucoma. However, the utility of structural measurements in moderate and advanced stages of the disease remains unclear. There is evidence that RNFL and optic disc assessment by imaging technologies may not provide adequate sensitivity to follow patients who present with severe glaucomatous damage. [22]–[26] In this situation, visual field injury may still be the best method to quantify the impact of the disease and monitor its progression. It follows that for optimal diagnosis and management of glaucoma, clinicians should consider measures that are from both structural and functional domains. We recently proposed a method for estimating RGC loss from a combination of SAP and RNFL assessment with optical coherence tomography (OCT). [10], [11], [27] The method takes advantage of the different performance of structural and functional tests according to the stage of the disease. It obtains a final estimate of RGC count for an individual eye, which is a combined estimate of the estimates from the structural and the functional test, but weighted for disease severity. The combined RGC estimates performed significantly better than isolated structural and functional parameters for staging the disease and monitoring progression.

Our study has limitations. We used empirically derived formulas to estimate the number of RGCs from OCT data and our estimates of RGC counts were not based on direct histological RGC counts in humans. It could be argued that our observations are just the result of the empirical formulas used to obtain RGC counts; however, several pieces of evidence give support to our method. The empirical formulas derived by Harwerth and colleagues [9] have been validated on histological studies in monkeys that have a visual system almost indistinguishable to that of humans. The relationship between predicted RGC counts and histological measured RGC numbers had an R^2^ of 0.9, indicating an almost perfect predictive value. Therefore, if the empirical formulas closely predict the histological counts, there is little reason to believe that our findings are just an artifact from the calculations. We used Stratus OCT to evaluate RNFL measurements. Newer versions of this technology such as spectral domain OCT provide higher resolution images with better reproducibility compared to time domain OCT. [28]–[31] However, as the empirical formula for estimation of RGC counts was developed based on studies using time domain OCT, we wanted to apply the same technology in our study.

In conclusion, this study revealed limitations of VFI as a surrogate for neural losses in glaucoma. Our findings suggest that although VFI was designed to account for the higher density of RGCs in the central fovea, it underestimates the amount of neural loss in glaucoma, especially in early to moderate disease. Additionally, as the relationship between VFI and estimated number of RGCs is nonlinear, disease severity should be taken into account when interpreting rates of VFI change over time.
